# Local coronary wall eccentricity and endothelial function are closely related in patients with atherosclerotic coronary artery disease

**DOI:** 10.1186/s12968-017-0358-2

**Published:** 2017-07-06

**Authors:** Allison G. Hays, Micaela Iantorno, Michael Schär, Monica Mukherjee, Matthias Stuber, Gary Gerstenblith, Robert G. Weiss

**Affiliations:** 1 Department of Medicine, Division of Cardiology, Johns Hopkins University, 600 N Wolfe St., Baltimore, MD 21287 USA; 2 Department of Radiology, Division of Magnetic Resonance Research, Johns Hopkins University, 600 N. Wolfe St., Baltimore, MD 21287 USA; 3 Department of Radiology, Centre Hospitalier Universitaire Vaudois, Center for Biomedical Imaging (CIBM), University of Lausanne, Lausanne, Switzerland

**Keywords:** Coronary artery disease, Endothelium, Magnetic resonance imaging

## Abstract

**Background:**

Coronary endothelial function (CEF) in patients with coronary artery disease (CAD) varies among coronary segments in a given patient. Because both coronary vessel wall eccentricity and coronary endothelial dysfunction are predictors of adverse outcomes, we hypothesized that local coronary endothelial dysfunction is associated with local coronary artery eccentricity.

**Methods:**

We used 3 T coronary CMR to measure CEF as changes in coronary cross-sectional area (CSA) and coronary blood flow (CBF) during isometric handgrip exercise (IHE), a known endothelial-dependent stressor, in 29 patients with known CAD and 16 healthy subjects. Black-blood MRI quantified mean coronary wall thickness (CWT) and coronary eccentricity index (EI) and CEF was determined in the same segments.

**Results:**

IHE-induced changes in CSA and CBF in healthy subjects (10.6 ± 6.6% and 38.3 ± 29%, respectively) were greater than in CAD patients 1.3 ± 7.7% and 6.5 ± 19.6%, respectively, *p* < 0.001 vs. healthy for both measures), as expected. Mean CWT and EI in healthy subjects (1.1 ± 0.3 mm 1.9 ± 0.5, respectively) were less than those in CAD patients (1.6 ± 0.4 mm, *p* < 0.0001; and 2.6 ± 0.6, *p* = 0.006 vs. healthy). In CAD patients, we observed a significant inverse relationship between stress-induced %CSA change and both EI (r = -0.60, *p* = 0.0002), and CWT (r = -0.54, *p* = 0.001). Coronary EI was independently and significantly related to %CSA change with IHE even after controlling for mean CWT (adjusted r = -0.69, *p* = 0.0001). For every unit increase in EI, coronary CSA during IHE is expected to change by -6.7 ± 9.4% (95% confidence interval: -10.3 to -3.0, *p* = 0.001).

**Conclusion:**

There is a significant inverse and independent relationship between coronary endothelial macrovascular function and the degree of local coronary wall eccentricity in CAD patients. Thus anatomic and physiologic indicators of high-risk coronary vascular pathology are closely related. The noninvasive identification of coronary eccentricity and its relationship with underlying coronary endothelial function, a marker of vascular health, may be useful in identifying high-risk patients and culprit lesions.

## Background

Endothelial-dependent coronary artery vasoreactivity is an important indicator of vascular function and a predictor of future cardiovascular events [1–3]. Endothelial dysfunction in the coronary arteries is believed to play a critical role in the development and progression of local coronary atherosclerosis. Coronary endothelial function (CEF) is heterogeneous and varies among coronary segments in a given patient with coronary artery disease (CAD) for reasons that are incompletely understood [2, 4]. Likewise, coronary atherosclerosis is also a spatially-heterogeneous process with the extent and characteristics of atheroma varying extensively within a given CAD patient. Plaque eccentricity is common and is significantly related to features of lesion vulnerability and an increased risk of adverse clinical sequelae [5–7]. However, the assessment of both coronary functional and anatomic indices of disease traditionally required invasive techniques and therefore their relationship has not been well characterized in healthy and low-risk or stable populations [1, 8].

Both anatomic and functional changes of the coronaries can now be measured non-invasively using cardiovascular magnetic resonance imaging (CMR). Early anatomic changes associated with the development of atherosclerosis include outward arterial remodeling with preservation of the luminal size [9]. Because coronary vessel wall thickening precedes luminal narrowing, the degree of CAD may be underestimated with conventional imaging of the coronary lumen using x-ray angiography, CT or CMR [10]. However, early indices of coronary vessel wall remodeling including increased coronary wall thickness can be identified and reproducibly quantified using black blood CMR techniques [11–14], thereby enabling non-invasive detection and characterization of arterial remodeling and subclinical coronary atherosclerosis.

Likewise, coronary endothelial function (CEF), which is impaired early in the atherosclerotic process and is a predictor of subsequent cardiovascular events [2, 3, 15], can also be measured using non-invasive CMR methods. Recent studies demonstrate that CMR measures of CEF performed during isometric handgrip exercise (IHE) quantify nitric-oxide mediated coronary endothelial vasoreactivity [16] with excellent short- and longer-term reproducibility [17, 18].

Although invasive studies indicate that plaque composition may play an important role in atherosclerotic disease and coronary endothelial dysfunction [19], the underlying anatomic features of the coronary vessel wall (increased thickness and eccentricity) and their relationship to an early initiator of atherosclerotic disease, endothelial dysfunction, are not well characterized. Defining the presence of both in the same arterial segment, therefore, may enhance the non-invasive identification of those segments which are most vulnerable to the development and progression of disease. Moreover, because both plaque eccentricity and coronary endothelial dysfunction are associated with adverse outcomes [2, 3, 20], we used noninvasive CMR techniques to test the hypothesis that local coronary endothelial dysfunction is associated with local plaque eccentricity in patients with known CAD and in healthy age-matched subjects.

## Methods

### Participants

All participants provided written informed consent to a protocol approved by the Johns Hopkins School of Medicine Institutional Review Board. Subjects were outpatients at Johns Hopkins with no contraindications to CMR. Healthy subjects had no history of CAD or traditional CAD risk factors and, those over age 50 years had a <10 Agatston coronary artery calcium score on computed tomography (CT)[21] . CAD subjects were individuals without unstable coronary syndrome for at least 6 months, who were asymptomatic (no anginal symptoms) at the time of the study, and with a prior clinically indicated coronary x-ray angiography or coronary CTA (computed tomography angiography) documenting >30% luminal stenosis in at least one vessel. The data reported in this study are not a subset of prior published reports. Participants underwent CMR between February 2013 and March 2016.

### Study protocol

CMR was performed on a commercial human 3 T CMR scanner (Achieva, Philips Healthcare, Best, NL) in the morning after an overnight fast. For endothelial function imaging, alternating anatomical and velocity-encoded images were collected at baseline and during approximately 5 min of continuous isometric handgrip exercise (IHE) as previously described [17, 22, 23]. Cross-sectional anatomical [24] and flow velocity encoded spiral CMR [25] were obtained using single breath-hold cine sequences [22, 26] with reproducibility of the techniques previously published [17, 18]. CMR parameters were: the temporal/spatial resolution for the anatomic images was: 15 ms/0.89×0.89×8.0 mm^3^ and 34 ms/0.8×0.8×8 mm^3^ for the flow velocity images (velocity encoding = 35 cm per second). Approximately 15–24 cardiac phases were acquired for the coronary flow scan, depending on heart rate. Radiofrequency excitation angle = 20°, 17 spiral interleaves were acquired, and all scans were prospectively triggered. The imaging plane for both endothelial function and wall thickness measurements was localized in a proximal or mid coronary segment that was straight, without branches and no more than mild luminal stenosis (≤30% luminal stenosis) over a distance of approximately 2 cm (based on prior coronary x-ray angiography or coronary CTA that was clinically indicated, and confirmed by visual assessment of 3D coronary MRA used to plan the 2D slices). In several cases, more than one 2D imaging plane was prescribed per subject. Black blood coronary vessel wall imaging was performed at rest using dual-inversion spiral imaging with a heart rate-dependent inversion time [27]. CMR parameters were: echo time = 0.84 ms, spectrally selective fat suppression, breath-hold duration approximately 15 to 24 s, acquisition window 21 ms, spatial resolution (acquired/reconstructed) **=** 0.6 × 0.6 × 8.00 mm^3^/0.49×0.49×8.00 mm^3^ and a radiofrequency excitation angle = 45°. Linear volume shimming, localized to the intersection of the imaging slice and the heart, was performed to minimize off-resonance effects on spiral imaging; the readout durations of spiral interleaves were 13, 30, and 22 ms for anatomic, flow, and black blood sequences respectively. The total duration of the CMR exam was approximately 40–45 min. Heart rate and blood pressure were measured throughout the study using ECG and a noninvasive, CMR-compatible blood pressure monitor (Invivo, Orlando, FL). The rate pressure product (RPP) was calculated as systolic blood pressure x heart rate.

### Image analysis

**Fig. 1:**
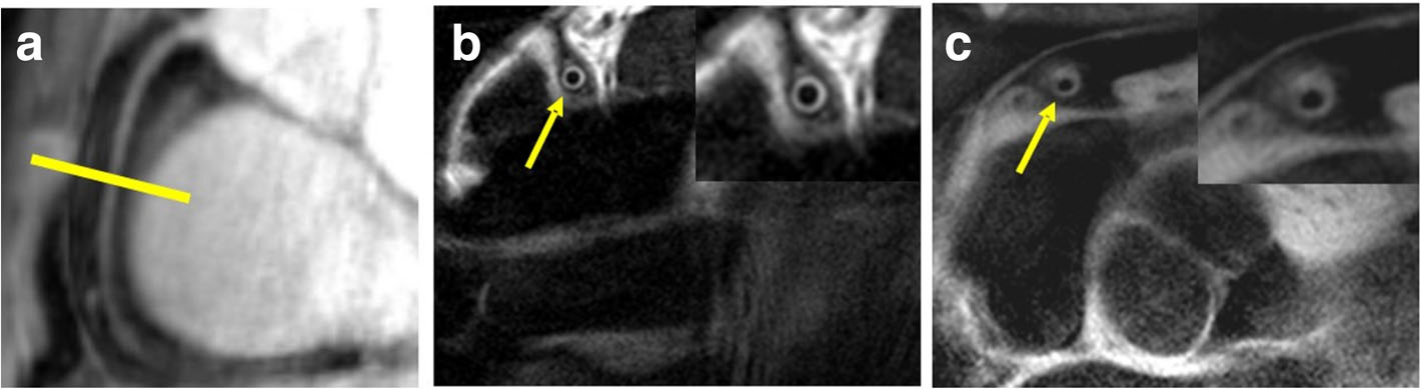
CMR example of coronary black blood vessel wall imaging. Cross-sectional images in a young, healthy volunteer and in a patient with mild CAD. **a** A scout scan obtained along the right coronary artery (RCA) in a healthy subject is shown together with the location for cross-sectional imaging (*yellow line*). *Yellow arrows* denote RCA cross section. **b** The normal volunteer has an eccentricity index (maximum wall thickness/minimum wall thickness) of 1.3. **c** The CAD patient has an eccentricity index of 3.3, indicating eccentric arterial remodeling

Images were analyzed for cross-sectional area (CSA) using a semi-automated software tool as previously reported [16, 22]. Semi-automated measurements of black-blood coronary cross sectional images were made (Soap Bubble version 4.5) to determine minimum, mean, and maximum coronary wall thicknesses (CWT) and eccentricity index (EI) (=the ratio of maximum to minimum CWT, Fig. 1) [28, 29]. For coronary flow velocity (CFV) and coronary blood flow (CBF) measurements, images were analyzed using semi-automated commercial software [22, 30].

Additional analyses were performed in all subjects to determine whether the combined effect of abnormal EI and abnormal CWT was associated with significantly greater impairment in CEF than was either variable alone. Coronary segments with both abnormal EI (defined as EI >1 SD above the mean EI of the healthy subjects) *and* abnormal mean CWT (defined as CWT >1 SD above the healthy mean CWT) had CEF quantified and compared with subjects with either abnormal EI alone, those with abnormal CWT alone, and those with neither abnormal CWR nor abnormal EI.

### Statistical analysis

Data are expressed as mean values ± one standard deviation. The data were tested for normality using the Shapiro-Wilk test. Parametric (Student’s t-test) and non-parametric testing (Wilcoxon signed rank test for paired data and Wilcoxon rank sum test for non-paired data) were used as appropriate. Linear regression analysis was performed to assess the relationship between continuous variables of coronary vasoreactivity and the anatomic atherosclerosis indices of coronary wall measurements. Multiple linear regression was employed to ascertain the relationship between EI and CEF after controlling for CWT (STATA version 13). Statistical significance was defined as a two-tailed *p*-value <0.05.

## Results

All participants completed the study without complication. A total of 31 arterial segments in 29 CAD patients and 19 segments in 16 healthy subjects were analyzed. Baseline characteristics of the study population are presented in Table 1.

**Table 1:** Characteristics of the subjects

	Healthy subjects (*N* = 16)	CAD patients (*N* = 29)	*p-value*
Age – years			
Mean ± SD	57 ± 21.2	58 ± 14.8	0.86
Range	25–80	37–83	
Male --no. (%)	8 (50)	21 (67)	0.37
Left Ventricular Ejection Fraction -- %	N/A	59 ± 5.2	
Coronary artery imaged: Total segments	19	31	
RCA alone --no. (%)	11 (69)	20 (69)	NS
LAD alone--no. (%)	3 (19)	7 (24)	NS
Both RCA and LAD --no. (%)	2 (12)	2 (7)	NS
History of prior angioplasty	N/A	9 (31)	
History of CABG	N/A	1 (3)	
Significant multivessel CAD (>50% luminal stenosis in major epicardial coronary artery)	N/A	7 (24)	
Body Mass Index (Mean ± SD) (BMI, kg/m^2^)	26.0 ± 5.2	27.1 ± 5.9	0.49
CAD risk factors^a^ Mean ± SD	0	1.3 ± 0.8	<0.001
ACE-inhibitor use --no. (%)	0	12 (39)	<0.001
Beta-blocker use --no. (%)	0	15 (48)	<0.001
Statin use --no. (%)	0	20 (69)	<0.001

*Abbreviations*: *N/A* not applicable, *SD* standard deviation, *CAD* coronary artery disease, *RCA* right coronary artery, *LAD* left anterior descending artery, *ACE* angiotensin converting enzyme inhibitor, *NS* non-significant

^a^CAD risk factors excluding age and gender

### Hemodynamic effect of isometric handgrip stress

Isometric handgrip exercise (IHE) induced significant hemodynamic changes in both healthy subjects and patients. We observed a 30.1 ± 17.6% increase in the rate pressure product (RPP) with IHE stress in the healthy subjects and a 32.8 ± 17.2% increase in the CAD patients (*p* < 0.0001 stress vs. baseline, both groups). The absolute RPP during stress and the percent increase in RPP from baseline did not differ between the healthy subjects and CAD patients (*p* = 0.48, *p* = 0.6 respectively).

### Coronary vasoreactivity

**Fig. 2:**
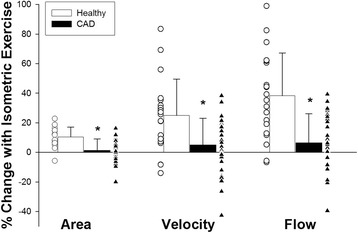
Changes in coronary area, peak flow velocity, and blood-flow during IHE. Individual data points of relative changes in coronary vasoreactive parameters with IHE are shown for healthy subjects (*open circles*, *n* = 19 coronary segments) and CAD patients (b*lack triangles*, *n* = 31 coronary segments) for coronary area, velocity and flow change in response to IHE. Adjacent to individual data points are bars showing mean +/- SD. There were statistically significant differences between healthy and CAD patients in all three vasoreactive parameters

Baseline coronary cross-sectional area (CSA) was similar in healthy subjects and in patients with CAD (13.2 ± 4.5 mm^2^ vs. 13.7 ± 5.8 mm^2^, healthy vs CAD, *p* = 0.8). Coronary arteries dilated significantly more with IHE stress in the healthy subjects than those of CAD patients in that the percent change in stress-induced CSA was significantly higher in healthy age matched subjects (10.6 ± 6.6%) than in those with CAD (1.3 ± 7.7%, *p* < 0.0001, Fig. 2), consistent with prior reports [17, 22].

### Coronary flow velocity and blood-flow measures

Peak diastolic CFV at baseline was similar in healthy subjects and patients with CAD (12.8 ± 3.8 cm/s vs. 14.0 ± 6.0 cm/s, healthy vs CAD, *p* = 0.40). The relative exercise-induced change in CFV was also greater in healthy subjects (+25.0 ± 24.7%) than in CAD patients (5.1 ± 17.9%, *p* = 0.001, Fig. 2). Likewise, baseline CBF was similar in healthy and CAD subjects (50.4 ± 19.2 ml/min vs 57.5 ± 31.7 ml/min, *p* = 0.39) but the CBF change with IHE stress was also significantly greater in the healthy group (+38.3 ± 29%) than in the CAD group (+6.5 ± 19.6%, *p* = 0.0001). Figure 2 illustrates relative changes (%) in coronary area, velocity and flow with IHE in both groups.

### Coronary wall measurements

**Fig. 3:**
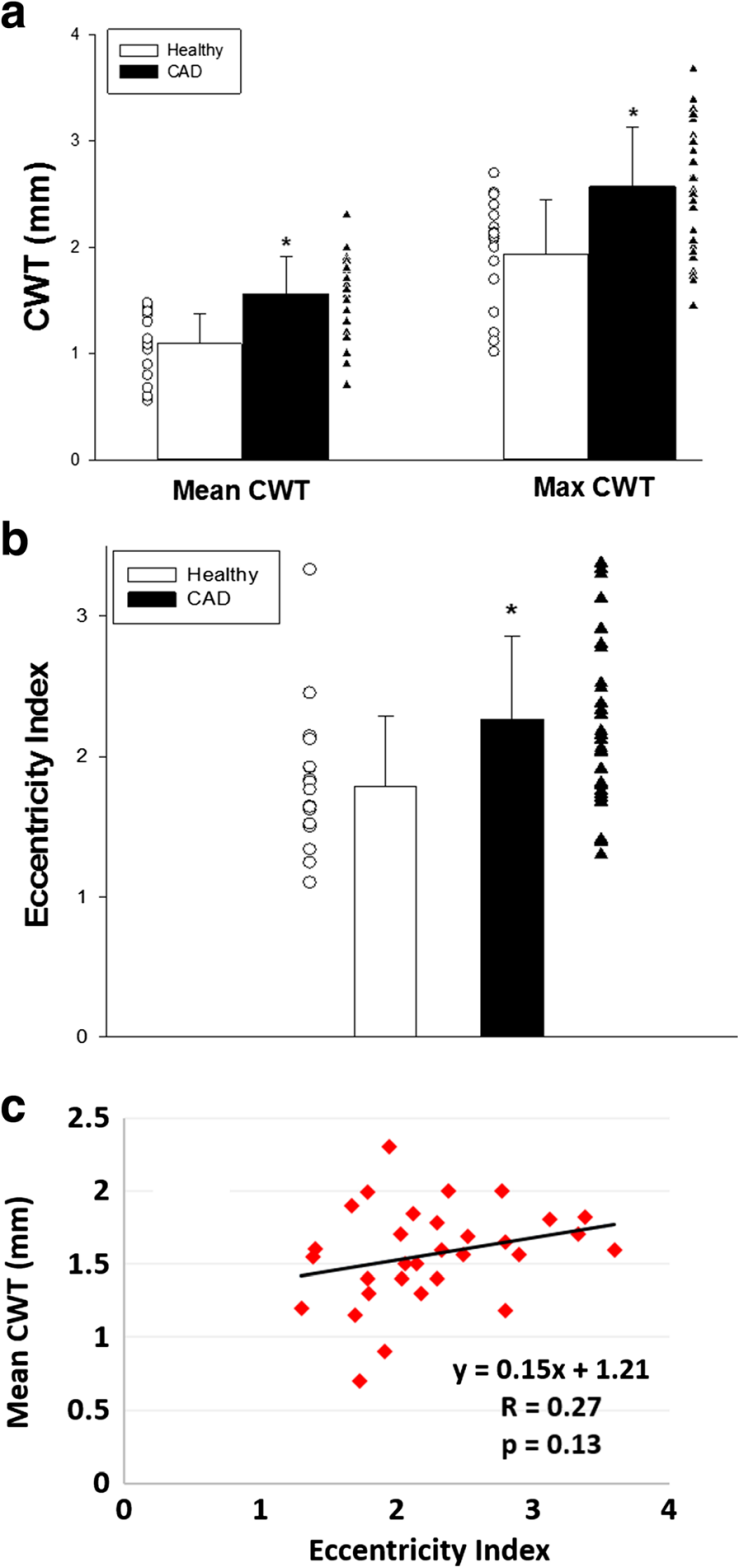
Coronary wall measurements in CAD patients and healthy subjects. **a** Individual data points (and mean +/- SD) showing mean and maximum coronary wall thickness (CWT, in mm) for healthy individuals (*open circles*) and CAD patients (*black triangles*, * *p* < 0.0001 vs. healthy for both measures). **b** Individual data points (and mean +/- SD) showing coronary eccentricity index (EI, defined as ratio: maximum CWT/minimum CWT). EI is significantly higher in CAD patients than in healthy age-matched adults. * *p* = 0.006 vs. healthy. **c** Relationship between mean CWT and EI in CAD patients using regression analysis

Mean and maximum coronary wall thicknesses were 1.1 ± 0.3 mm and 1.9 ± 0.5 mm, respectively in the healthy subjects, and mean EI was 1.8 ± 0.5 in this group. Both mean and maximum CWT were higher in CAD patients (1.6 ± 0.4 mm and 2.6 ± 0.6 mm respectively *p* < 0.0001 healthy vs. CAD for both measures). Similarly, mean EI was significantly higher in the CAD group (2.3 ± 0.6, *p* = 0.006 vs. healthy group, Fig. 3) compared to the healthy group.

### Relationship between coronary artery remodeling and endothelial-dependent coronary vasoreactivity

In healthy subjects, there was no significant relationship between measures of coronary wall remodeling (mean CWT or EI) and measures of CEF (Table 2). However, in CAD patients we observed a significant inverse relationship between mean CWT and %CSA change with stress (r = -0.54, *p* = 0.001, Fig. 5). Importantly, there was a significant inverse relationship between EI and %CSA change with IHE (r = -0.60, *p* = 0.0002, Fig. 5) in CAD patients. There was no significant relationship between CFV and either remodeling parameter (mean CWT and EI). Although we observed an inverse relationship between %CBF change and CWT in CAD patients, this was of borderline significance (r = -0.34, *p* = 0.05). There was no significant relationship between %CBF and EI (r = -0.27, *p* = 0.13, Fig. 5). Because mean CWT and EI were each inversely related to %CSA change with IHE (Fig. 5), we employed regression analysis to control for mean CWT and found that a significant relationship persisted between EI and %CSA change (adjusted r = -0.69, *p* = 0.0001), indicating that EI is independently associated with depressed (or impaired) CEF. For every unit increase in EI, coronary CSA with IHE is expected to decrease by -6.7 ± 1.7% (95% confidence interval: -10.3 to -3.0, *p* = 0.001). Using regression analysis, there was no significant relationship between the two coronary wall indices (mean CWT and EI, R = 0.27, *p* = 0.13, Fig. 3c). Furthermore, no significant interaction was detected between the variables CWT and EI (*p* value for interaction = 0.81).

**Table 2:** Relationship between coronary anatomic and functional measures

% Coronary Vasoreactive Change with IHE vs. Coronary Wall Measure:	% area change	% velocity change	% flow change
Healthy: Mean Coronary Wall Thickness	*R* = -0.17	*R* = 0.02	*R* = -0.02
	*P* = 0.46	*P* = 0.93	*P* = 0.92
Healthy: Eccentricity Index	*R* = 0.18	*R* = 0.08	*R* = 0.14
	*P* = 0.46	*P* = 0.74	*P* = 0.56
CAD: Mean Coronary Wall Thickness	*R* = -*0.54*	*R* = -0.11	R = -0.34
	*P* = *0.001*	*P* = 0.51	P = 0.05
CAD: Eccentricity Index	*R* = -*0.60*	*R* = -0.06	*R* = -0.27
	*P* = *0.0002*	*P* = 0.75	*P* = 0.13

Relationship between MRI measures of coronary remodeling (Coronary eccentricity index and mean coronary wall thickness) vs. coronary endothelial functional measures (% area change, % coronary peak velocity change and % coronary blood flow change with IHE) in healthy subjects and in patients with coronary artery disease using regression analysis

Italized items indicate statistically significant relationships

**Fig. 4:**
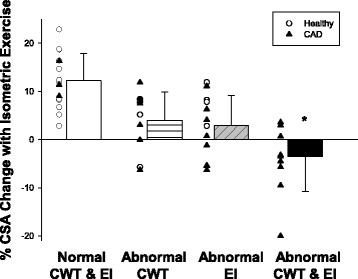
Coronary area change with isometric handgrip stress in four groups. Individual data bars (mean +/- SD) are shown for the following groups: 1) Normal coronary wall thickness (CWT) and eccentricity index (EI) defined as values within one standard deviation of that of healthy subjects’ values (*white bar*). 2) Coronary segments with abnormal CWT defined as CWT ≥1 standard deviation from the mean CWT of the healthy group (*white bar*, *horizontal lines*) 3) Segments with abnormal eccentricity index (EI, defined as EI ≥ 1 SD above the mean of the healthy group (*gray bar*, *diagonal lines*) and 4) Segments with *both* abnormal EI and abnormal CWT (*black bar*), illustrating that coronary endothelial function is more impaired in subjects with both abnormal CWT and abnormal EI than in those with either finding alone. Individual data points in *open circles* signify healthy subjects and those in *black triangles* signify CAD patients. * *p* < 0.05 (vs. Abnormal CWT group) and *p* < 0.05 (vs. Abnormal EI group)

To determine whether the combined effect of abnormal EI (defined as EI > 2.2 which is >1 SD above the healthy mean EI) and abnormal CWT (defined as CWT > 1.37 mm which is >1 SD above the healthy mean CWT) was associated with significantly greater impairment in CEF than either variable alone, we observed that coronary segments with both abnormal EI *and* abnormal mean CWT (*n* = 10 segments) had significantly reduced CEF (measured by %CSA change during IHE) as compared with subjects with abnormal EI alone (*n* = 13), those with abnormal CWT alone (*n* = 12), or neither (*n* = 15, for total of 50 segments in 29 CAD patient and 16 healthy subjects, Fig. 4).

## Discussion

The current study demonstrates the novel finding that an eccentric pattern of coronary wall remodeling is significantly and independently associated with abnormal local CEF in patients with mild CAD, and therefore that anatomic and physiologic early indicators of coronary vascular pathology are closely related. More specifically, coronary endothelial-dependent vasoreactivity was quantified with CMR-IHE testing and demonstrated a strong inverse relationship between coronary macrovascular endothelial function, as measured by IHE-induced %CSA change, and the degree of local coronary wall eccentricity in patients with mild CAD. There was no relationship between the two indices in healthy control subjects. We previously reported that coronary endothelial function is closely related to the degree of coronary luminal stenosis and local coronary wall thickening [17, 22]. Although increased mean CWT and EI tend to occur in CAD patients and with aging, the two variables were not significantly related to one another in this population in the mild, non-stenotic vessels studied (Fig. 3). This novel noninvasive CMR approach combines functional (CEF) and anatomical imaging of the coronaries that were previously shown to be reproducible in the short and longer term [13, 14, 17, 18, 22], and may provide important insights in the pathophysiology of early atherosclerotic coronary disease.

The values for coronary wall thickening and coronary endothelial function reported here are similar to those previously reported using CMR [11, 31] and invasive techniques [2, 32, 33], although with different endothelial-dependent stressors. The values for CEF are similar to those obtained in our prior CMR studies, demonstrating a coronary vasodilatory response to IHE in the healthy subjects, and no vasodilatory response or a vasoconstrictor response in many CAD patients [16, 17, 22]. We previously showed that the administration of nitroglycerin, an endothelial independent stressor, caused normal vasodilatation in coronary segments with mild to severe CAD that exhibited abnormal responses to IHE. The finding that nitroglycerin dilated the same coronary artery segments in CAD patients that constricted by IHE demonstrated that endothelial-independent mechanisms were intact and, by inference, that the mechanism responsible for the impaired IHE-related coronary response in CAD is abnormal endothelial function rather than a mechanical disturbance such as may occur with heavy coronary calcification or increased vascular stiffness [17]. Although it is possible that severely calcified vessels may have mechanical properties that could limit dilatation, in the current paper we did not study patients with severe CAD and only studied segments with no more than mild CAD. Further, these findings confirm prior observations that mean coronary wall thickness is significantly higher in CAD patients than in healthy subjects which, in the presence of preserved luminal area, is indicative of positive arterial remodeling [9, 11]. We extend measures of wall eccentricity from other vascular beds [34, 35] to the coronary arteries and show that eccentricity of the coronary arteries of CAD patients with no significant luminal stenosis is greater than that of healthy age-matched individuals.

**Fig. 5:**
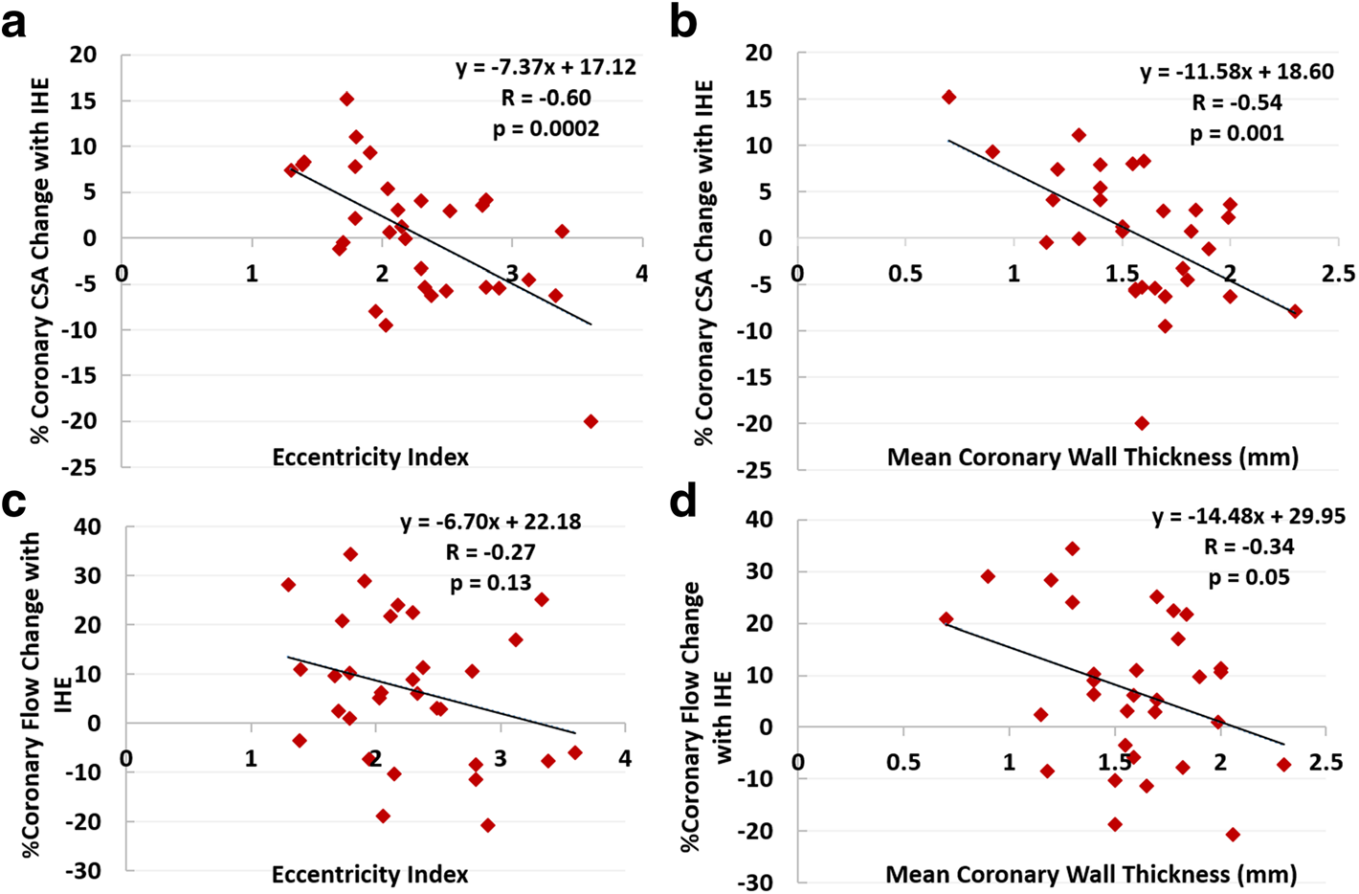
CMR measures of coronary remodeling indices vs. measures of coronary endothelial function in CAD patients (*N* = 31 arterial segments in 29 subjects)**. a** Coronary eccentricity index versus % coronary cross sectional area (CSA) change with isometric handgrip exercise (IHE). **b** Mean coronary wall thickness (CWT) versus %CSA change with IHE. **c** Coronary eccentricity index versus % coronary flow change with IHE. **d** Mean coronary wall thickness (CWT) versus % coronary flow change with IHE

Although our study demonstrated a relationship between coronary macrovascular changes in CSA and coronary wall eccentricity index in CAD patients (Fig. 5a), there was no significant relationship between IHE-induced coronary velocity or flow change and EI in either the CAD or the healthy group (Fig. 5c,d). These findings suggest that *local* increased eccentricity is more closely related to measures of *local* coronary endothelial function (e.g. epicardial area change in a given coronary segment) rather than to more global endothelial function measures (velocity and flow) that also reflect changes in downstream microvascular vasoreactivity, distant from the eccentricity.

We previously reported that abnormal coronary endothelial function varied among arteries in a given CAD patient and was related to the presence and degree of coronary wall thickening [22]. The observations here confirm that finding in different patients (Fig. 5) but also extend it, demonstrating that not only the degree but also the type of coronary wall remodeling (eccentric vs. concentric) is associated with impaired local coronary endothelial function in early coronary disease. Advances in CMR hardware (i.e. multitransmit radiofrequency) and software allow improved visualization of the coronary vessel wall, permitting a more detailed analysis of wall remodeling than was previously possible. The type of coronary wall remodeling (concentric vs. eccentric) may play an important role in local plaque progression or acute coronary syndrome [36]. Prior studies report that acute coronary syndromes tend to occur in vessels with angiographically mild disease [6]. One recent study determined that the presence in the coronary vessel wall of thin cap fibroatheromas (detected on intravascular ultrasound (IVUS)) and plaque burden are associated with an increased risk of acute coronary syndrome in the same vessel [37]. Non-invasive studies of coronary plaque composition may also play an important role in risk prediction [38, 39]. Therefore, based on these and other studies, the type and degree of coronary vessel remodeling and plaque composition [19] are important local factors that may potentially lead to unstable coronary syndromes.

Endothelial dysfunction is both a cause and a consequence of atherosclerosis, and thus, in terms of the correlation between local abnormal CEF and eccentricity observed here, it is difficult to determine whether depressed CEF contributes to coronary wall thickening/eccentricity, vice versa or both. It is clear that local factors contribute to the focal development of atherosclerosis and that local differences in three-dimensional coronary blood flow affect endothelial shear stress which, in turn, both modulates endothelial cell function and biochemistry [40] and drives plaque formation [41]. Eccentric lesions are associated with inflammation and are prone to rupture, which results in clinical events [35]. Others have suggested that changes in eccentricity over time may also be a strong predictor of future plaque rupture [40] and this noninvasive CMR approach is well suited to serial studies over time as well.

Recently, coronary endothelial function, eccentricity, wall shear stress, and plaque burden were measured invasively in patients with chest pain referred for angiography [42]. Wall shear stress was directly related to macrovascular CEF and inversely related to EI. Segments with the most plaque burden and lowest wall shear stress manifested impaired CEF and greater plaque eccentricity [42]. Unfortunately in that IVUS study the authors did not report whether there was a relationship between eccentricity and depressed CEF, as studied here for the first time. Taken together, the prior invasive studies and the current noninvasive report demonstrate that abnormal CEF varies regionally throughout the coronary vasculature and is related locally to plaque characteristics (amount, composition, and eccentricity/geometry) that were previously associated with adverse outcomes. Because acute vascular events tend to occur in areas of eccentric atherosclerosis [20, 35], the identification of areas of wall eccentricity and the correlation with underlying coronary endothelial function may be critically important in identifying potential high risk culprit lesions.

One limitation to the current study is that CMR imaging of the coronaries cannot distinguish separate layers of the vessel wall or measure coronary plaque components due to limits of spatial resolution. Although IVUS and optical coherence tomography are more sensitive at detecting and characterizing early vessel wall disease than CMR, they are invasive. Although multi-detector computed tomography (MDCT) has been used to assess coronary remodeling and plaque [43], the exposure to ionizing radiation and contrast media limit its application in low risk populations and in repeated studies. In addition, MDCT cannot directly measure coronary velocity or flow for the complete assessment of CEF. Although we primarily focused on proximal and mid coronary segments to evaluate stress-induced area changes, the ability to also measure coronary velocity and flow permits a more global assessment of downstream endothelial function that complements the measures of local epicardial vasoreactivity. Now that the relationship between local CEF and eccentricity has been identified, future work is needed to expand these noninvasive measures to longer coronary artery segments and the circumflex artery distribution as well as long-term studies to relate these measures to subsequent outcomes in large populations. The current coronary scan coverage is limited and this merits further improvement. Nevertheless, these coronary CMR CEF methods enable reproducible, noninvasive measures of coronary vasoreactivity that are NO-dependent [16] and that have been reported in a wide range of healthy subjects and patients with coronary artery disease.

## Conclusions

In summary, the present findings demonstrate that the degree of coronary wall eccentricity is inversely related to local macrovascular CEF in patients with mild CAD. Moreover, the relationship between coronary eccentricity and local CEF is independent of coronary wall thickness. These findings indicate that an eccentric pattern of coronary wall remodeling is associated with abnormal local CEF, and therefore that anatomic and physiologic early indicators of coronary vascular pathology are closely related in early atherosclerotic disease. This novel imaging approach may offer important insights into the pathobiology of atherosclerosis, the local progression of CAD, and the identification of high-risk patients and possibly high-risk coronary lesions.

## Abbreviations

ACE: Angiotensin converting enzyme
BMI: Body mass index
CABG: Coronary artery bypass graft
CAD: Coronary artery disease
CBF: Coronary blood flow
CEF: Coronary endothelial function
CFV: Coronary flow velocity
CSA: Coronary cross sectional area
CTA: Computed tomography angiography
CWT: Coronary wall thickness
EI: Eccentricity index
IHE: Isometric handgrip exercise
IVUS: Intravascular ultrasound
LAD: Left anterior descending
MDCT: Multi-detector computed tomography
NO: Nitric oxide
RCA: Right coronary artery
RPP: Rate-pressure product
